# Effect of Different Silane Coupling Agents In-Situ Modified Sepiolite on the Structure and Properties of Natural Rubber Composites Prepared by Latex Compounding Method

**DOI:** 10.3390/polym15071620

**Published:** 2023-03-24

**Authors:** Zhanfeng Hou, Dawei Zhou, Qi Chen, Zhenxiang Xin

**Affiliations:** Key Laboratory of Rubber-Plastics, Ministry of Education, Shandong Provincial Key Laboratory of Rubber-Plastics, School of Polymer Science and Engineering, Qingdao University of Science and Technology, Qingdao 266042, China

**Keywords:** natural rubber (NR), sepiolite, silane coupling agent, crosslink network structure, dynamic and static properties

## Abstract

With the increasing demand for eco-friendly, non-petroleum-based natural rubber (NR) products, sepiolite, a naturally abundant, one-dimensional clay mineral, has been identified as a suitable material for reinforcing NR through the latex compounding method. To create superior NR/sepiolite composites, three silane coupling agents with different functional groups were used to modify sepiolite in situ via grafting or adsorption during the disaggregation and activation of natural sepiolite, which were subsequently mixed with natural rubber latex (NRL) to prepare the composites. The results showed that the modified sepiolite improved the dispersion and interfacial bonding strength with the rubber matrix. VTES-modified sepiolite containing C=C groups slightly improved the performance but retarded the vulcanization of the NR composites, and MPTES and TESPT-modified sepiolites containing -SH and −S_4_− groups, respectively, effectively accelerated vulcanization, inducing the composites to form a denser crosslink network structure, and exhibiting excellent dynamic and static properties, such as the modulus at a 300% increase from 8.82 MPa to 16.87 MPa, a tear strength increase from 49.6 N·mm^−1^ to 60.3 N·mm^−1^, as well as an improved rolling resistance and abrasive resistance of the composites. These findings demonstrate that modified sepiolite can be used to produce high-quality NR/sepiolite composites with enhanced properties.

## 1. Introduction

Natural rubber is a highly elastic material that is obtained from the natural latex of rubber trees through a series of processing steps [1]. It is widely used in various fields such as tire production, transmission, and transportation [2,3]. To enhance its performance and reduce costs, reinforcing fillers are added to natural rubber. Carbon black [4,5] and silica [6,7] are the most commonly used reinforcing materials in the rubber industry. However, the production of carbon black relies on non-renewable petroleum-based energy sources, while silica production consumes a significant amount of energy and causes environmental pollution [8]. To address these challenges and promote sustainability, researchers have sought alternative materials, such as new structured carbon-based materials such as graphene [9] and carbon nanotubes [10,11], bio-based materials such as cellulose [12,13], and clay mineral materials [14,15,16] such as montmorillonite and kaolin. Clay mineral materials are of particular interest due to their abundant reserves, easy accessibility, and low cost. Moreover, clay minerals have diverse structural forms, such as two-dimensional lamellar montmorillonite [17] and kaolinite, as well as one-dimensional fibrous sepiolite and palygorskite [18,19], which can achieve high levels of reinforcement after appropriate treatment. Therefore, they have become a popular choice for enhancing the performance of natural rubber.

Sepiolite is a naturally abundant, non-toxic, one-dimensional fibrous silicate material that has an ideal structural formula of Si_12_O_30_Mg_8_(OH)_4_(H_2_O)_4_∙8H_2_O [20], with fiber lengths ranging from 0.2 µm to 5 µm [21]. Its unique structure comprises two continuous tetrahedral sheets and one discontinuous octahedral sheet, which create many tunnels and channels, leading to a large specific surface area. Sepiolite also has a high density of silanol groups, which form as a result of the combination between non-shared oxygen atoms of the tetrahedral silicon sheets and hydrogen [22]. Sepiolite’s special fibrous crystal morphology, large specific surface area, and abundant silanol groups give it excellent adsorption, enhancement, and stable suspension in the aqueous phase. However, natural sepiolite fibers tend to exist more as aggregates or bundles due to van der Waals forces between fibers. Moreover, complex mineral formation conditions often lead to the co-existence of trace-associated minerals with sepiolite, reducing its specific surface area and surface activity [23,24]. These factors can limit its dispersion in the polymer matrix and limit its application as a nanomaterial.

To achieve excellent natural rubber/sepiolite composites, two key challenges in their processing and application must be addressed: the dispersibility of sepiolite within the rubber matrix and the strength of the interfacial bonding between sepiolite and natural rubber [25]. By enhancing the dispersion of sepiolite in the natural rubber matrix and leveraging the intrinsic properties of sepiolite and natural latex, these composites can be prepared using the economical and environmentally friendly latex compounding method [26,27]. This approach [28] offers several advantages over traditional melt mixing, including lower energy consumption, reduced dust pollution, and improved filler dispersion in the rubber matrix, resulting in an enhanced composite performance. In the latex compounding method, sepiolite must be disaggregated and activated to prepare homogeneous sepiolite dispersions. The most effective approach to achieve this involves ultrasonic disaggregation combined with acid-thermal activation, such as that of Ruiz-Hitzky [29,30], who successfully prepared a highly stable sepiolite suspension system using ultrasonic means, while Jiménez-López [31] and Zhou et al. [23] employed thermal activation by HNO_3_ and microwave-assisted thermal activation by HCl, respectively, leading to a more significant increase in the specific surface area and surface activity of sepiolite. To improve the strength of the sepiolite–polymer interface, researchers have used various approaches. Hayeemasae [32] utilized sepiolite-reinforced epoxidized natural rubber, leveraging strong interactions between sepiolite’s hydroxyl and siloxane groups and epoxy groups. Raji [33] and Peinado [34] modified sepiolite with aminosilanes and added it to polypropylene and poly(lactic acid), respectively, to enhance the compatibility of sepiolite with polymers and material properties. Silane coupling agent-modified sepiolite, as used by Wang et al. [35] to reinforce cis-polybutadiene rubber, significantly improved the mechanical properties of the resulting composites, particularly when KH560 was used at 7%, which increased tensile and tear strengths by 108.3% and 74.1%, respectively. These results suggest that the addition of a silane coupling agent has a substantial impact on improving the strength of the sepiolite–polymer interface.

Previous research [32,36,37] has primarily focused on melt mixing, and there is limited information on in situ modification of sepiolite for latex compounding. In this study, we selected three silane coupling agents with different functional groups (see Table 1) to modify sepiolite. Our modification mechanism [38,39,40] involved the hydrolysis of Si–O–C_2_H_5_ in the silane coupling agent and the subsequent condensation of hydroxyl groups on the surface of sepiolite, with C=C in VTES, -HS- in MPTES, and –S_4_– in TESPT all able to participate in the vulcanization process of natural rubber and form strong chemical bonds. Hydrolysis of silane coupling agents is known to be slow, often requiring the addition of acid to promote hydrolysis [41,42] and improve the efficiency of hydroxyl condensation with the inorganic filler surface. Capitalizing on the acidic conditions of sepiolite during depolymerization activation, we employed a one-step activation modification method to prepare in situ silane-modified sepiolite. This method not only improved the efficiency of sepiolite modification but also reduced energy and acid consumption. Morphology, activity, and modification levels of the modified sepiolite were characterized using X-ray diffraction (XRD), Fourier-transform infrared spectroscopy (FTIR), scanning electron microscopy (SEM), and thermogravimetric analysis (TGA). Next, we prepared natural rubber/sepiolite composites by emulsion mixing of the in situ modified sepiolite with natural latex, evaluating the dispersibility and interfacial binding ability using tensile section morphology, DSC, and bound rubber content. The effects of modified sepiolite on the vulcanization characteristics and the dynamic and static properties of the composites were also analyzed.

## 2. Experimental Materials and Methods

### 2.1. Materials

A thirty-six percent total solid content of low-ammonia NRL was obtained from the Chinese Academy of Tropical Agricultural Sciences (Danzhou, China). Sepiolite concentrate (X-ray fluorescence (XRF) chemical analysis indicated that the composition of this fraction is 65.19% SiO_2_, 20.25% MgO, 7.89% Al_2_O_3_, 3.11% Fe_2_O_3_, 1.05% CaO) was obtained from Qingdao Zhongxiang Environmental Protection Technology Co., Ltd (Qingdao, China). The silanes, namely Triethoxyvinylsilane (VTES; 97%; M_w_ = 190.31 g/mol), (3-Mercaptopropyl)Triethoxysilane (MPTES; 98%; M_w_ = 238.42 g/mol), and Bis[3-(Triethoxysilyl)Propyl]Tetrasulfide (TESPT; 90%; M_w_ = 538.95 g/mol), were purchased from Shanghai Macklin Biochemical Technology Co., Ltd. (Shanghai, China). Oxalic acid dihydrate (OA, C_2_H_2_O_4_·2H_2_O, AR), zinc oxide (ZnO), and stearic acid (SA) were purchased from Sinopharm Chemical Reagent Co., Ltd. (Shanghai, China). The N-cyclohexyl benzothiazole-2-sulphonamide (Accelerator CZ), 2,2′-dibenzothiazoledisulfde (Accelerator DM), and sulfur (S) were industrial grade and provided by SanLux Co., Ltd. (Shaoxing, China).

### 2.2. Preparation of Sepiolite Dispersions

To begin, 20 g of sepiolite powder was added to an aqueous solution at a solid-to-liquid ratio of 1:20 (*w*/*w*). The mixture was then sonicated using a TiAl-V tip sonicator (SCIENTZ JY99-IIDN, with a 22 mm diameter tip, Ningbo, China) in pulses of 5 s on and off for a total of 12 min to achieve a homogeneous suspension. Next, 6 g of oxalic acid was added to the sepiolite suspension, and the mixture was stirred at 80 °C for 6 h. The pH of the suspension was then adjusted to a range between 3.5 and 4.5 by rinsing with deionized water. After this, 2 g of VTES, MPTES, or TESPT silane coupling agents was added to the suspension and stirred for an additional 4 h at 80 °C. The suspension was then washed with deionized water until it reached a pH of 7, resulting in the in situ modified sepiolite with a silane coupling agent. The modified sepiolite samples were named VTES-Sep, MPTES-Sep, and TESPT-Sep, while a comparison sample was synthesized using only oxalic acid and neutral pH washing, and was named Sep.

To obtain part of the pure sepiolite and modified sepiolite powder, the corresponding modified sepiolite slurry was dried. The sepiolite powders were extracted using ethanol in a Soxhlet extractor for 24 h, with 15 min reflux intervals, to remove any un-grafted silane coupling agent. Finally, the extracted sepiolite powders were dried in an oven at 80 °C for 24 h and were prepared for characterization using XRD, FTIR, and TGA.

### 2.3. Preparation of Sep/NR Masterbatches and Composites

The NRL was diluted with deionized water to a concentration of 20 wt%. Sepiolite suspensions were then separately mixed with NRL by stirring at 500 rpm for 30 min, with a mass ratio of 20% sepiolite to NR (e.g., 20 g dry weight of sepiolite for every 100 g dry weight of NR). The mixture was then flocculated with a 2% CaCl_2_ solution, washed with water, and dried in a vacuum oven at 60 °C to obtain sepiolite/NR masterbatches.

**Figure 1:**
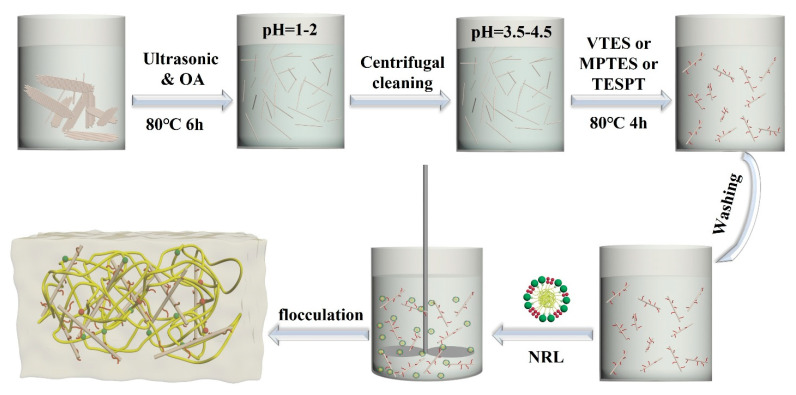
Schematic diagram of sepiolite modification and the latex compounding method.

The masterbatches were plasticized eight times in a two-roll open mill. Then, the ingredients for vulcanization and other additives were added one-by-one to the masterbatch, with a total mixing time of 10 min. The compounds were then vulcanized using an XLR-D vulcanizer at 150 °C under a pressure of 10 MPa for the optimum cure time (t90), as determined using a non-rotor curemeter. After curing, the samples were air-cooled to obtain the composites. The composites are coded as Sep/NR, VSep/NR, MSep/NR, and TSep/NR. The process of sepiolite modification and latex compounding is shown in Figure 1, and the formulation of sepiolite/NR compounds is demonstrated in Table 2.

### 2.4. Characterizations

XRD images of the sepiolites were obtained by a Rigaku D-MAX 2500-PC diffractometer (Tokyo, Japan) with nickel-filtered Cu Kα radiation of λ = 0.154 nm. The scanning rate was 5°/min, and the test angle was 5–70°.

FTIR spectra of sepiolite were recorded on a Bruker VERTEX 70 spectrometer (Bruker Optik GmbH Co., Ettlingen, Germany) by averaging 32 scans at a 4 cm^−1^ resolution, with the wavenumber ranging from 4000 to 400 cm^−1^.

TGA was used to evaluate the thermal degradation of sepiolite and the impact of silane functionalization on sepiolite. The analysis was carried out using a STA449(F5) Thermogravimetric Analyzer (NETZSCH-Gerätebau GmbH, Selb, Germany). To accurately determine the number of silane graft modifications, a representative sample was heated in a platinum pan under air from room temperature to 800 °C, with a heating rate of 10 °C/min. The amount of grafted and intercalated silane molecules has been calculated using the following equation [33,43]:

(1) $$\mathrm{grafted}\;\mathrm{amount}\;(\mathrm{mequiv}/g)\;=\;\frac{10^{3}W_{150-650}}{(100-W_{150-650})M}$$

where W_150–650_ is the number of silane degradation between 150 and 650 °C, and M (g/mol) is the molecular weight of the grafted silane molecules.

The surface morphology of sepiolites and tensile fractured surfaces of vulcanizates were observed by SEM performed on a JSM-7500F (JSOL, Tokyo, Japan). All specimens were sputtered with gold before observations.

The bound rubber content [25] was measured on un-vulcanized compounds. Firstly, 0.5 g of the un-vulcanized compounds were cut into small pieces and put into a sample cage prepared by nickel mesh (400 mesh). Then, the sample cage was placed in a frosted glass bottle with toluene and immersed for 72 h at room temperature. The toluene was replaced every 24 h. Lastly, the residual sample was taken out from the toluene and dried at 80 °C in a vacuum to a constant weight. Three samples of each group were tested, and the average was taken as the final result.

The bound rubber content was calculated according to the following equation:

(2) $$\mathrm{Bound}\;\mathrm{rubber}\;\mathrm{content}\;=\;\frac{M_{1}-M_{0}\times f}{M_{0}-M_{0}\times f}$$

where *M*_0_ is the initial weight of the sample, *M*_1_ is the mass of the sample dried to constant weight, and *f* is the weight fraction of sepiolite in the compound.

The specific heat capacity curves were acquired through the differential scanning calorimeter test (DSC, NETZSCH-204, NETZSCH, Selb, Germany). The samples were performed at a 10 °C/min heating rate at −100–25 °C in a nitrogen atmosphere. The normalized specific heat capacity step (∆*C_pn_*) and the mass fraction of the immobilized polymer layer (*χ_im_*) were calculated as follows [44,45]:

(3) $$∆C_{pn}=∆C_{p}/(1-w)$$

(4) $$\chi_{im}=\frac{∆C_{p0}-∆C_{pn}}{∆C_{p0}}$$

where ∆*C_p_* is the heat capacity jump at T_g_ and was obtained by the software NETZSCH Thermal Analysis, *ω* is the weight fraction of the filler, and ∆*C_p_*_0_ indicates the specific heat capacity variation at T_g_ of unfilled NR.

Curing characteristics were evaluated using a rotorless rheometer (MDR, Alpha Technologies, Akron, OH, USA) at 150 °C, and the Flory–Rehner equation [46] was used to determine the crosslinking density based on the equilibrium swelling method with toluene as the solvent. Toluene has a solubility parameter (18.2) similar to that of natural rubber (16.2–17.0). Three measurements were conducted for each sample, and the mean values with statistical errors are presented.

Tensile tests, stress relaxation experiments, and tear tests were conducted on a Zwick Roell material testing machine (Z005, Zwick/Roell GmbH Co., Ulm, Germany). Type 2 dumbbell samples prepared according to ISO37-2005 [47] were used to perform the stress relaxation and stress–strain tests at 25 °C. Stress–strain curves were obtained by carrying out simple uniaxial tension tests at an extension rate of 500 mm/min. The stress relaxation curves were recorded at a constant strain of 100% for 1000 s. Tear tests were performed on angle test pieces (approximately 100 × 20× 2 mm^3^) at an extension rate of 500 mm/min, following the ISO 34-1-2015 standard [48]. Five measurements were conducted for each sample, and the average value with statistical errors is reported.

The strain-dependent storage modulus (G′) and the loss factor (tanδ) of the rubber compounds were analyzed using a rubber process analyzer (RPA2000, Alpha Technologies, Akron, OH, USA). The strain amplitude changed from 0.28% to 100% at the test frequency of 1 Hz and a temperature of 60 °C.

The dynamic mechanical analysis (DMA) was carried out in a tension mode on a Dynamic Thermomechanical Analyzer (DMTS, EPlexor 500N, NETZSCH-Gerätebau GmbH, Selb, Germany). The dumbbell samples of type 2 with dimensions of ca. 75 × 4 × 2 mm^3^ (ISO-37-2005) [47] and a test length of 10 mm were cut from the vulcanizate sheets. The measurements were performed at temperatures between −80 °C and 80 °C with a heating rate of 3 °C/min at a dynamic strain of 0.1%, a static strain of 0.5%, and a frequency of 10 Hz.

Abrasive resistance was evaluated by a DIN abrader (GT-7012D, GOTECH Testing machines Co., Ltd., Taiwan, China) with a standard of ISO 4649-2017 [49]. The reported values were averaged from three independent results of volume loss.

## 3. Results and Discussion

### 3.1. Characterization of Pure and Modified Sepiolite

**Figure 2:**
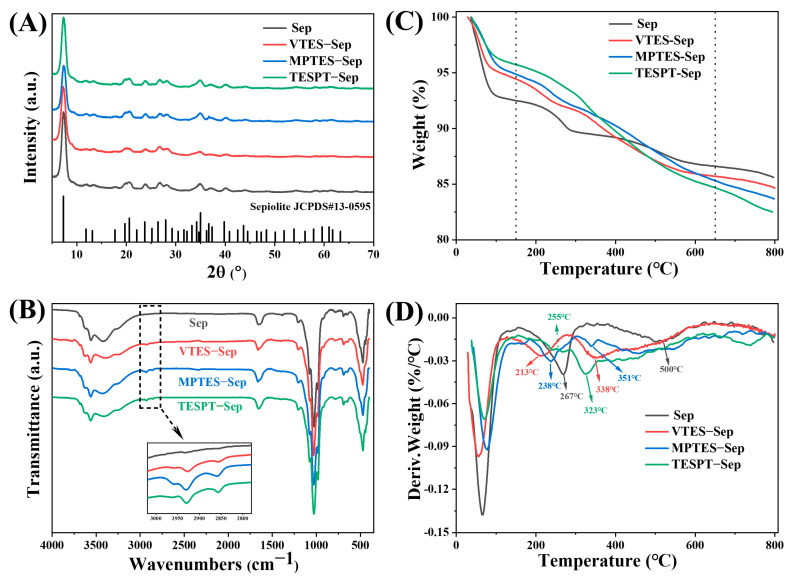
Characteristics of pure and modified sepiolite. (**A**) XRD patterns, (**B**) FTIR spectra, (**C**) TGA curves, and (**D**) derivative TGA curves.

XRD analysis was utilized to investigate any changes in the crystal structure of sepiolite. Figure 2A illustrates the XRD patterns of sepiolite and silane-modified sepiolite. Upon comparison with the standard JCPDS map for sepiolite, it was found that all reflections of unmodified sepiolite were consistent with it, and no additional diffraction peaks were detected. This indicates that the purity of sepiolite was improved after undergoing disaggregation activation treatment. The XRD patterns of the silane-modified sepiolite were similar to those of unmodified sepiolite, with characteristic reflections at 2θ = 7.3° (d = 12.1 Å), 20.6° (d = 4.31 Å), and 35.0° (d = 2.56 Å). This observation confirms that the silane modification does not alter the crystal structure of sepiolite, consistent with prior findings by Tartaglione et al. [50].

In Figure 2B, the FTIR spectra of sepiolite and silane-modified sepiolite are shown. Sepiolite contains various types of water molecules, including adsorbed water, zeolitic water, bound water, and structural water, which are present inside the channels or on the surface [51]. The stretching vibrations of (Mg/Al)−OH groups and Si−OH groups of sepiolite were assigned to absorption bands at 3626 cm^−1^ and 3526 cm^−1^, respectively [52]. The stretching vibration of −OH, primarily from surface-adsorbed water and zeolitic water, resulted in a broad band centered at 3420 cm^−1^. The H−O−H bending vibration of zeolitic water and bound water led to the appearance of a band at 1660 cm^−1^. The antisymmetric stretching vibration and stretching vibration of the Si−O−Si group of the tetrahedral sheets caused the bands at 1204 cm^−1^ and 1026 cm^−1^, respectively [51]. Additionally, the appearance of new bands at 2929 and 2850 cm^−1^ in VTES−Sep, MPTS−Sep, and TESPT−Sep, corresponding to the antisymmetric and symmetric stretching vibration of C−H in organosilanes, respectively, indicates the successful grafting of VTES, MPTS, or TESPT onto sepiolite [53].

The thermogravimetric (TG) and derivative thermogravimetric (DTG) curves of sepiolite and silane-modified sepiolites are presented in Figure 2C,D, and their weight losses are summarized in Table 3. The thermogravimetric curves of unmodified sepiolite displayed four discrete weight losses. The initial weight loss before 150 °C was attributed to the evaporation of adsorbed water and zeolite water [54] (i.e., adsorbed on the external surface and in the structural channels), with a loss of 7.5%. Although, this value is not entirely consistent with the literature [54,55] and is mainly related to the environmental humidity and the hydrophilicity of sepiolite. The elimination of bound water occurred in two stages [56], from 150 °C to 400 °C and 400 °C to 650 °C, with maximum weight loss temperatures of 267 °C and 500 °C, respectively, resulting in a total weight loss of 5.9%. As the temperature increased, the hydroxyl groups in the sepiolite condensed and dehydrated, ultimately leading to complete structural damage, with a loss of 1.02% between 650 °C and 800 °C. The weight loss of silane-modified sepiolite was reduced up to 150 °C, indicating that the hydrophilicity of the modified sepiolite was reduced. DTG analysis in Figure 2D revealed that the volatilization of the modifier on modified sepiolite was divided into two distinct steps. The first step occurred at a relatively low temperature (T < 267 °C), and the weight loss of VTES−Sep and MPTS−Sep was more pronounced. This result is attributed to sepiolite having excellent adsorption properties, where due to its porous structure and the abundance of silanol groups on its surface, hydrolyzed silane molecules are easily adsorbed on sepiolite through hydrogen bonding or van der Waals forces [50]. The second step occurred at a relatively high temperature and was dominated by the volatilization of the grafted modifier [33]. The amount of silane modification was calculated based on the volatilization mass between 150 °C and 650 °C, indicating that the percentage of silane molecule grafted on sepiolite was about 2.83% for VTES-Sep, 3.66% for MPTS−Sep, and 5.16% for TESPT−Sep. Furthermore, the number of intercalated molecules that effectively participated in the silylation reaction was estimated to be 0.282 mequiv/g for VTES-Sep, 0.251 mequiv/g for MPTS-Sep, and 0.120 mequiv/g for TESPT−Sep. These results provide compelling evidence for the successful silylation of sepiolite.

**Figure 3:**
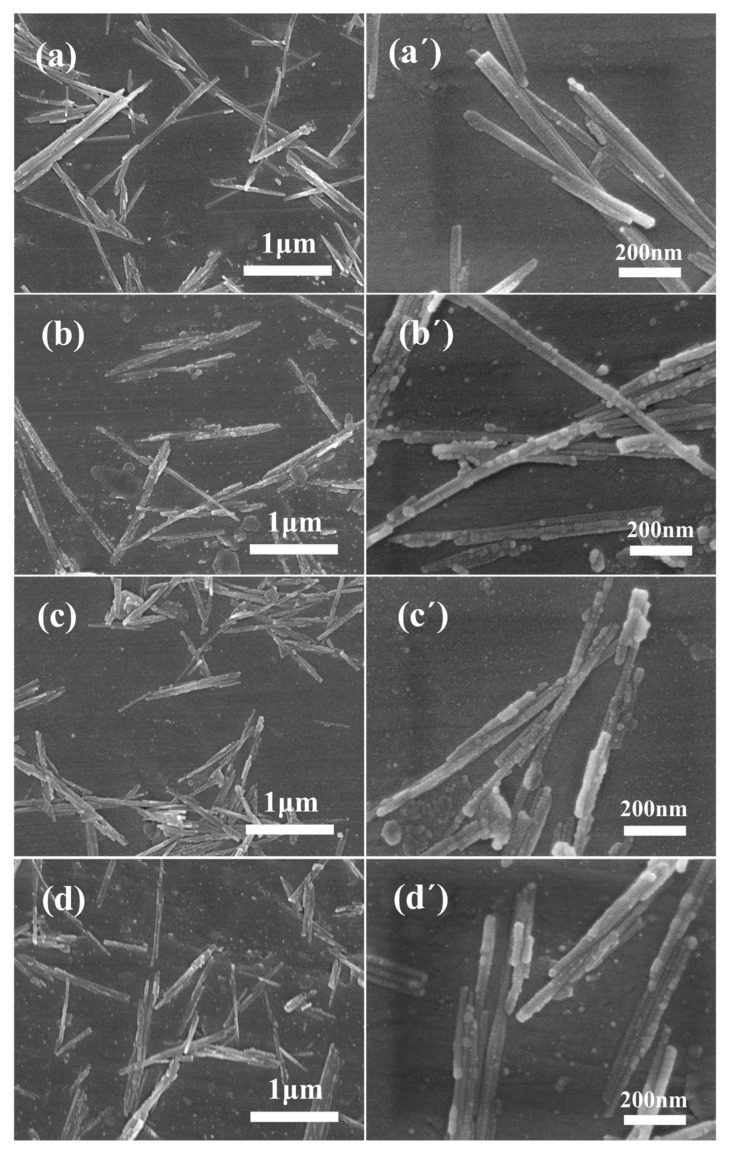
Microscopic morphology of sepiolite and silane-modified sepiolites. (**a**,**a′**) Sep, (**b**,**b′**) VTES−Sep, (**c**,**c′**) MPTES−Sep, and (**d**,**d′**) TESPT−Sep (left ×20,000, right ×100,000).

SEM images (Figure 3) were used to investigate the microstructure and morphological changes of sepiolite, with the sample being prepared by adding a diluted suspension of sepiolite to the sample table, followed by drying and gold sputtering. The SEM images revealed that the shape structure and aggregation morphology of sepiolite before and after modification remained largely unchanged, with rod and micro-bundle shapes being observed in all cases (Figure 3a–d). At a magnification of 10,000 times (Figure 3a′–d′), it was observed that the surface of unmodified sepiolite was relatively smooth, whereas the surfaces of sepiolite modified by silane coupling agents displayed distinct changes. Specifically, sepiolite modified by VTES exhibited a greater number of spherical protrusions, similar in shape to those observed in sepiolite modified by VTMS [57], and the surfaces of sepiolite modified by MTPS and TESPT showed more coverage and roughness. These observations indicate that all three silane coupling agents successfully graft onto the surface of sepiolite.

### 3.2. Dispersion and Interfacial Interaction of Sepiolite/NR Composites

**Figure 4:**
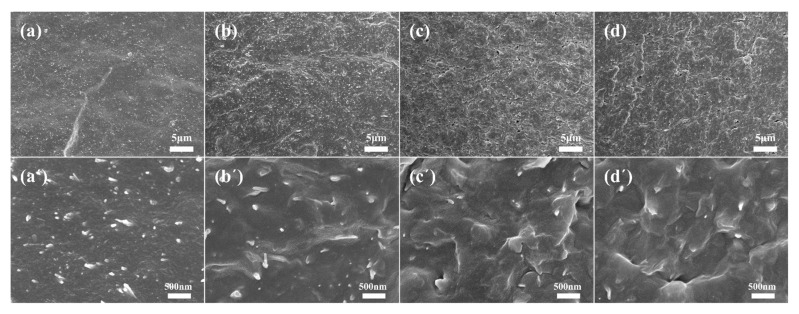
SEM micrographs of tensile fractured surfaces of (**a**,**a’**) Sep/NR composite, (**b**,**b’**) VSep/NR composite, (**c**,**c’**) MSep/NR composite, and (**d**,**d’**) TSep/NR composite.

In order to visually characterize the dispersion and interfacial interactions of sepiolite in natural rubber, we employed SEM to observe the morphology of the tensile fracture surface of vulcanized rubber. Figure 4a,a’ reveals that the fracture surface of the Sep/NR vulcanizates is relatively smooth, with some sepiolite rod-like particles visibly exposed on the surface, and a small amount of particles aggregated. This suggests that while the wet compounding process can improve sepiolite dispersion in the matrix, unmodified sepiolite exhibits lower compatibility with the natural rubber matrix. In contrast, Figure 4b–d,b’–d’ depict the cross-sections of VSep/NR, MSep/NR, and TSep/NR vulcanizates, respectively. It can be seen that sepiolite particles are distributed in the natural rubber matrix as individual rods without any apparent aggregation, and most of the rod-shaped particles are embedded in the rubber matrix. Especially in MSep/NR and TSep/NR composites, sepiolite almost fused with the natural rubber, resulting in a blurred interface. These observations suggest that silane-modified sepiolite has better compatibility with the natural rubber matrix and can be more effectively dispersed within the rubber matrix, which improves the interfacial bond strength with the matrix, with MPTS and TESPT modifications demonstrating greater application efficacy than VTES modification.

**Figure 5:**
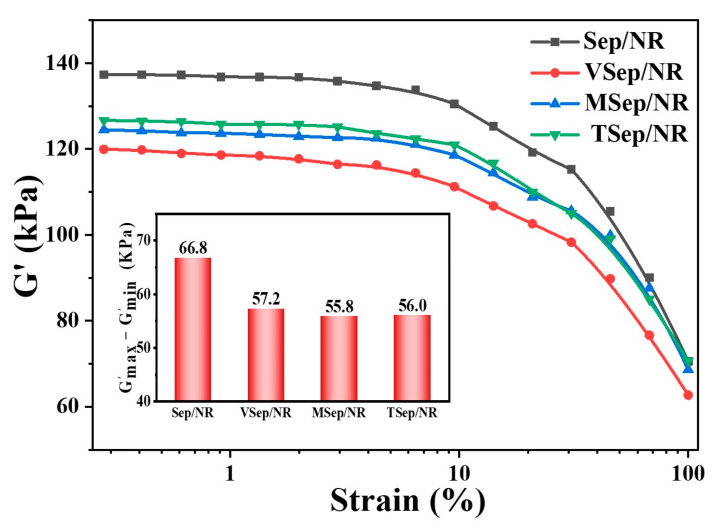
Strain amplitude dependence of the storage modulus (G’) of sepiolite/NR composites.

The storage modulus (G’) of uncured composites was measured as a function of strain amplitude and is shown in Figure 5. At low strains, all samples exhibited a rapid decrease in G’ with increasing strain amplitude, resulting in non-linear viscoelastic behavior known as the Payne effect [58]. This effect can be used to assess the filler network of the composites based on the difference between the maximum and minimum G’ (∆G’). The Sep/NR sample exhibited the strongest Payne effect, indicating a strong filler network and poor dispersion. The ∆G’ of the composites with silane-modified sepiolite showed a significant decrease, indicating an improvement in its dispersion in the rubber matrix and a reduction in the formation of its own filler network.

**Figure 6:**
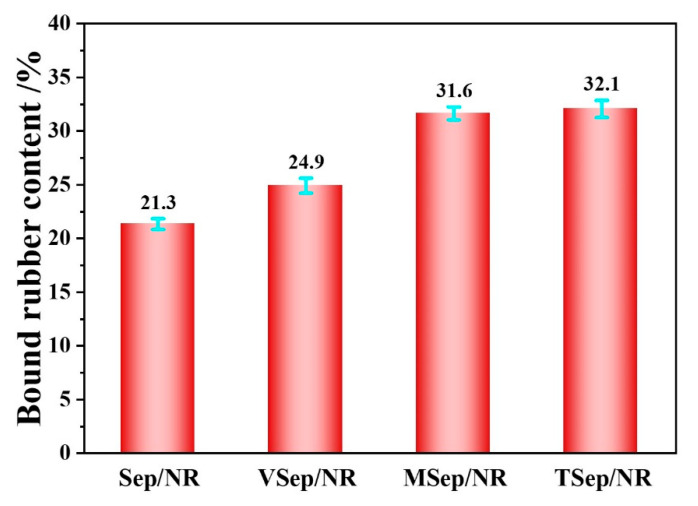
The bound rubber contents of sepiolite/NR composites.

The bound rubber content, which is defined as the indissoluble rubber in good solvents, can be used to characterize the interaction between the rubber and filler. The higher the bound rubber content, the stronger the interaction. Figure 6 displays the bound rubber content of all samples. The Sep/NR composites had the lowest bound rubber content at 21.3%, which was mainly due to the adsorption or entanglement of rubber molecular chains with the sepiolite. The composites with silane-modified sepiolite exhibited a significantly higher bound rubber content, with TSep/NR and MSep/NR similar at 32.1% and 31.6%, respectively, and VSep/NR at 24.9%. This is because the silane coupling agent is adsorbed and grafted onto the surface of sepiolite, which can entangle with more rubber molecular chains. Additionally, during the subsequent mixing process, due to the high local temperature, −S_4_− and -SH in TESPT−Sep and MPTES−Sep were activated and combined with rubber molecular chains to form chemical bonds, producing tightly bound rubber [59]. While C=C in VTES relies only on external sulfur addition to produce chemical bonding, the amount of bonded rubber formed is less due to the lower temperature of the applied sulfur.

**Figure 7:**
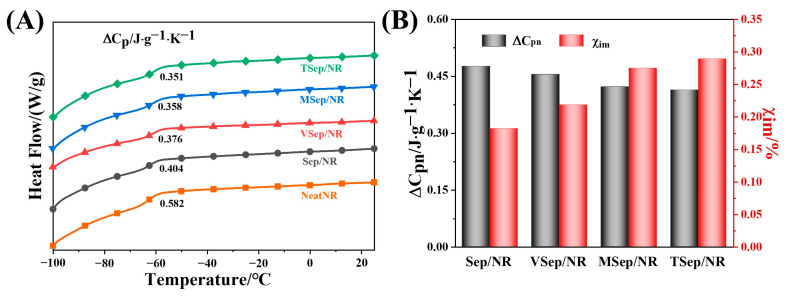
(**A**) DSC curves and (**B**) ∆*C_pn_* and χim, of sepiolite/NR composites.

The interfacial interaction between rubber and sepiolite can also be characterized by the mobility of rubber chain segments at and near the sepiolite particle surface. DSC was used to analyze the ∆*C_p_* of sepiolite/NR composites, as shown in Figure 7A, and the normalized specific heat capacity step (∆*C_pn_*) and mass fraction of the immobilized polymer layer (*χ_im_*) of sepiolite/NR composites are illustrated in Figure 7B. The values of ∆*C_p_* for NR/sepiolite composites were all lower than the Neat NR (the natural rubber contains zinc oxide, stearic acid, CZ, DM, and sulfur in the same amount as in Table 2, but without other reinforcing materials such as sepiolite), indicating restricted movement of molecular chains and chain segments due to the addition of sepiolite. Comparing the samples with silane coupling agents to Sep/NR samples, the ∆*C_p_* and ∆*C_pn_* values of the modified blends were significantly lower, and the mass fraction of the immobilized polymer layer (*χ_im_*) was significantly increased. This indicated tighter bonding between natural rubber and sepiolite and improved interfacial strength. Comparing VSep/NR, MSep/NR, and TSep/NR, it was observed that the *χ_im_* value of TSep/NR was the largest, followed by MSep/NR and VSep/NR, suggesting that there is a difference in the bonding ability between sepiolite and the rubber matrix after modification with different coupling agents. TESPT-modified sepiolite had the strongest bonding ability between sepiolite and the natural rubber matrix, and the interfacial strength was the largest, which was consistent with the change in the bound rubber content to the composite.

### 3.3. Vulcanization Characteristics of Sepiolite/NR Composites

**Figure 8:**
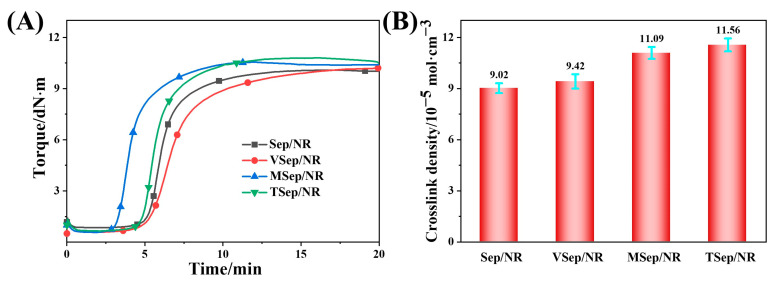
Characteristics of sepiolite/NR composites: (**A**) the curing curves and the (**B**) crosslinking density.

Curing is a crucial process in the production of rubber products. Figure 8A and Table 4 illustrate the curing characteristics of sepiolite/natural rubber composites. The scorch time (t10) represents the degree of early vulcanization. By increasing the scorch time, the occurrence of early crosslinking in linear molecules within the compound is reduced, leading to a lower likelihood of premature vulcanization. The optimum vulcanization time (t90) of the compound is shortened as the crosslinking speed of linear molecules is accelerated, resulting in a faster attainment of the maximum crosslinking density [60]. It is observed that the t10 and t90 of VSep/NR have increased compared to Sep/NR. This is because the functional group of VTES contains a C=C double bond, which increases the number of double bonds in the VTES-Sep and the natural rubber blend. As a result, the time required to achieve equilibrium crosslinking is prolonged. On the other hand, the t10 and t90 of MSep/NR and TSep/NR have decreased, indicating accelerated vulcanization rates. This is because the functional groups in MPTES and TESPT both contain −S−, which can participate in crosslinking reactions. The higher reactivity of -SH in MPTES resulted in a significantly faster vulcanization time of the composites. The minimum torque (M_L_) is related to the dispersion of the filler and the network structure in the compounded rubber. Table 4 shows that the M_L_ of all composites decreased compared to Sep/NR, suggesting that the silane modification of sepiolite improved its dispersion and restricted its network structure. The difference between the maximum torque and the minimum torque (M_H_−M_L_) represents the stiffness of the rubber composite, which is positively associated with the crosslinking density and the interaction between the filler and rubber. Table 4 and Figure 8B show that the M_H_-M_L_ and crosslinking density of the composites with modified sepiolite increased compared to Sep/NR. For VSep/NR composites, which rely solely on the higher dispersion of sepiolite, a slight increase in crosslinking density was shown [61], while for MSep/NR and TSep/NR, the presence of −S− groups in modified sepiolite can enhance the crosslinking density, where TESPT−Sep has the largest amount of grafting and the largest amount of −S−, resulting in the highest M_H_−M_L_ value and the highest crosslinking density [62].

### 3.4. Static Mechanical Properties of Sepiolite/NR Composites

**Figure 9:**
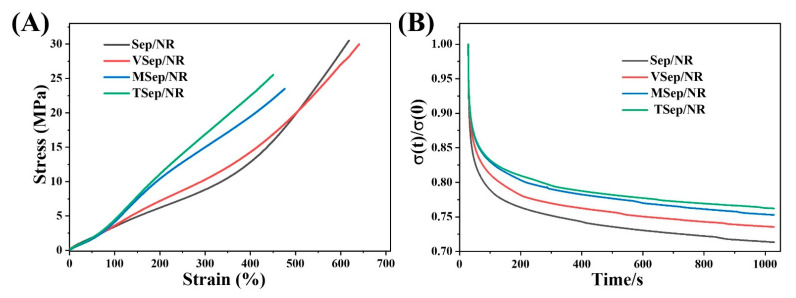
(**A**) The stress–strain curves of sepiolite/NR vulcanized composites, and (**B**) stress relaxation curves normalized with respect to the initial stress for vulcanizates (applied strain 100%).

Figure 9A illustrates the stress–strain behavior of sepiolite/NR composites, while Table 5 presents the corresponding static mechanical performance data. The static mechanical properties of VSep/NR were observed to have slightly improved compared to Sep/NR, which can be attributed to the improved dispersion of sepiolite. On the other hand, the tensile strength and elongation at break of MSep/NR and TSep/NR composites were found to decrease, but the modulus and tear strength were significantly enhanced. Specifically, the modulus at 300% of MSep/NR and TSep/NR increased from 8.82 MPa to 14.99 MPa and 16.87 MPa, respectively, representing an increase of 70% and 91%. Moreover, the tear strength increased from 49.6 N·mm^−1^ to 58.1 N·mm^−1^ and 60.3 N·mm^−1^, corresponding to a percentage increase of 17.1% and 21.6%, respectively. A higher modulus and tear strength are critical for certain dynamic applications of rubber products.

To gain a deeper understanding of the different network structures in the nanocomposites, the relaxation behavior of the nanocomposites under stress was characterized, as shown in Figure 9B. The stress relaxation curves of the four types of vulcanized rubbers under 100% strain for 1000 s were normalized by their respective initial stresses. All four vulcanizates displayed a typical stress relaxation behavior, where the stress rapidly decreased at the beginning and then decreased as the system approached equilibrium [63]. The stress relaxation of the Sep/NR composite was the largest, with the lowest stress value at equilibrium, owing to its low bound rubber content and fewer crosslinking networks. In contrast, the addition of silane-modified sepiolite to the composite resulted in better dispersion, a higher content of bound rubber, a stronger interfacial bonding ability, and a higher crosslinking density, resulting in a lower stress decrease and a higher equilibrium stress value. The trend of stress reduction for the composites modified with silane-modified sepiolites follows VSep/NR > Msep/NR > Tsep/NR, which is the opposite to the trend of bound rubber content and crosslinking density. This indicates the difference in network structures created by different silane-modified sepiolites within the composite.

**Figure 10:**
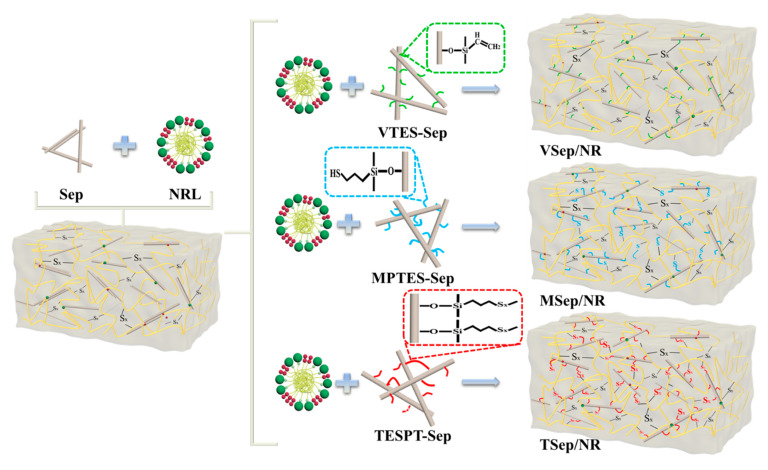
Schematic diagram of the structure of different modified sepiolite-reinforced natural rubbers.

As shown in Figure 10, the sepiolite grafted with VTES primarily consists of the C=C functional group, which relies on the addition of sulfur to form a chemical bond with the rubber molecular chain, as well as better dispersion, which increases the bound rubber content and crosslinking density. The sepiolite grafted with MPTES and TESPT not only has good dispersion, a large grafting molecular weight, and a more entangled rubber molecular chain, but can also form chemical bonds with rubber molecular chains via its own -HS and −S_4_− to form a denser bonded rubber and network crosslinking structure. Table 3 shows that TESPT-Sep grafting had the largest amount and the most -S- content, resulting in a more denser crosslinking network structure and stronger physical properties in TSep/NR composites.

### 3.5. Dynamic Properties of Sepiolite/NR Composites

**Figure 11:**
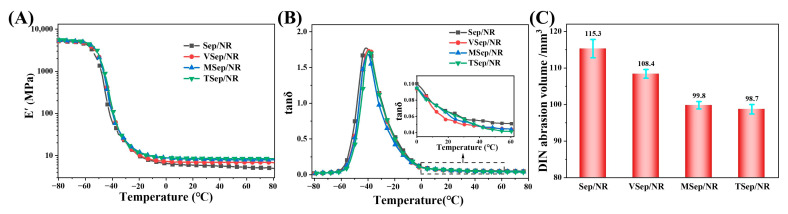
(**A**) Temperature dependence of the E’, (**B**) tan δ, and (**C**) DIN abrasion volume of sepiolite/NR composites.

The dynamic mechanical properties of a composite reflect the amount of energy stored as elastic energy and the amount of energy dissipated during strain, which are highly dependent on the volume fraction of the filler, its dispersion in the matrix, and the interfacial bonding between the filler and the matrix [64]. Figure 11A shows the temperature-dependent storage modulus (E’) of sepiolite/NR composites, which indicates that the addition of silane-modified sepiolite leads to higher E’. At 25 °C, the E’ values of TSep/NR and MSep/NR were 8.55 MPa and 8.18 MPa, respectively, representing an improvement of 45.4% and 39.1% over Sep/NR. This result suggests that silane modification induces stronger interfacial interactions between sepiolite and natural rubber matrix. Figure 11B shows the temperature dependence of loss factor (tan δ) of the sepiolite/NR composites, and all samples exhibited an obvious loss peak at around −40 °C, which corresponds to the glass transition temperature (T_g_) of the composites. The addition of silane-modified sepiolite led to a clear shift to higher T_g_ values, indicating that more polymer chains were grafted or adsorbed on the fillers, slowing down polymer kinetics and increasing the Tg of the composite. Additionally, the peak of tan δ tended to decrease due to the stronger interfacial interaction between the modified sepiolite and the rubber matrix and the more dense crosslinked network structure, which produced a larger E’ (tan δ = E”/E’).

In tire tread rubber, the tan δ at 0 °C and 60 °C are important parameters related to the wet skid resistance and the rolling resistance. High-performance rubber composites should have high tan δ 0 °C and low tan δ at 60 °C [65]. From Figure 11B, it is evident that the tan δ of composites with silane-modified sepiolite at both 0 °C and 60 °C were smaller than those of Sep/NR, indicating reduced wet skid resistance and improved rolling resistance. In addition, Figure 11C indicates that the DIN abrasion volume of composites with silane-modified sepiolite was reduced compared to Sep/NR, indicating improved abrasion resistance. In summary, the use of silane-modified sepiolite can improve the interfacial interaction between sepiolite and the rubber matrix, enhance the abrasion resistance, and improve the rolling resistance of the composite, but the wet skid resistance may be reduced.

## 4. Conclusions

This study presented an innovative in situ modification method for sepiolite to be used in latex compounding. Three types of silane coupling agents were successfully grafted onto the sepiolite surface during the disaggregation and activation of natural sepiolite, which was confirmed by FTIR, TG, and SEM analyses. This fabrication technique is both efficient and easy to operate. The silane coupling agents grafted onto the sepiolite surface promoted the dispersion of sepiolite in the natural rubber matrix and enhanced the interfacial bonding strength between sepiolite and natural rubber by increasing the entangled rubber molecular chains and chemical bonding interactions. VTES-modified sepiolite, which contains C=C, retarded the vulcanization and slightly improved the physical and mechanical properties of the composites. MPTES-modified sepiolite and TESPT-modified sepiolite, which both contain -S-, effectively accelerated vulcanization and led to a denser crosslinked network structure, resulting in stronger physical and mechanical properties of the composites. TESPT had the highest grafting amount, and the modulus at 300% increased from 8.82 MPa to 16.87 MPa, tear strength increased from 49.6 N·mm^−1^ to 60.3 N·mm^−1^, and the rolling resistance and abrasive resistance of the composites improved.

## Figures and Tables

**Table 1:** Structure and chemical characteristics of silane molecules.

Chemical Name	Functional Group	Structural Formula	M (g/mol)
Triethoxyvinylsilane (VTES)	vinyl	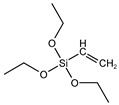	190.31
3-Mercaptopropyltriethoxysilane (MPTES)	mercapto	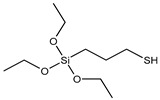	238.42
Bis[3-(triethoxysilyl)propyl] tetrasulfide (TESPT)	tetrasulfide	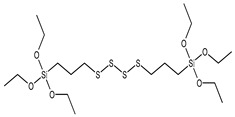	538.95

**Table 2:** Formulation of sepiolite/NR composites, phr ^a^.

Materials	Sep/NR	VSep/NR	MSep/NR	TSep/NR
Masterbatches	120 ^b^	120	120	120
VTES	0	2	0	0
MPTES	0	0	2	0
TESPT	0	0	0	2
zinc oxide	5	5	5	5
stearic acid	5	5	5	5
Accelerator CZ	2	2	2	2
Accelerator DM	1	1	1	1
sulfur	2	2	2	2

^a^ Parts per hundred of rubber. ^b^ 120 phr of the masterbatch = 100 phr of NR + 20 phr of sepiolite.

**Table 3:** Thermogravimetric analysis values of sepiolite and silane-modified sepiolites.

Materials	Weight Loss/%			Modifier/%	Grafted Amount/(mequiv/g)
	40–150 °C	150–650 °C ^a^	650–800 °C		
Sep	7.50	5.90	1.02	-	-
VTES-Sep	5.57	8.73	1.06	2.83	0.282
MPTES-Sep	5.13	9.56	1.66	3.66	0.251
TESPT-Sep	4.27	11.06	2.28	5.16	0.120

^a^ Silane modifier was evaluated between 150 °C and 650 °C.

**Table 4:** The curing characteristics of sepiolite/NR composites.

Samples	t10/min	t90/min	M_L_/dN∙m	M_H_/dN∙m	M_H_−M_L_/dN∙m
Sep/NR	5.29	8.89	0.84	10.09	9.25
VSep/NR	5.37	11.19	0.57	10.21	9.64
MSep/NR	3.33	6.94	0.57	10.55	9.98
TSep/NR	4.91	8.39	0.65	10.81	10.16

**Table 5:** Static mechanical properties of sepiolite/NR composites.

Samples	Modulus at 100%/MPa	Modulus at 300%/MPa	Tensile Strength/MPa	Elongation at Break/%	Tear Strength/N·mm^−1^
Sep/NR	3.45 ± 0.33	8.82 ± 0.88	30.0 ± 1.8	618 ± 58	49.6 ± 1.7
VSep/NR	3.61 ± 0.27	10.28 ± 0.91	29.98 ± 2.1	640 ± 47	50.3 ± 2.0
MSep/NR	4.14 ± 0.15	14.99 ± 0.53	23.4 ± 1.1	476 ± 35	58.1 ± 1.5
TSep/NR	4.43 ± 0.19	16.87 ± 0.62	25.5 ± 1.6	450 ± 36	60.3 ± 1.4

## Data Availability

The data that support the findings of this study are available from the corresponding author upon reasonable request.
